# A consensus on the definition of positive animal welfare

**DOI:** 10.1098/rsbl.2024.0382

**Published:** 2025-01-22

**Authors:** Jean-Loup Rault, Melissa Bateson, Alain Boissy, Björn Forkman, Bjørn Grinde, Lorenz Gygax, Jes Lynning Harfeld, Sara Hintze, Linda J. Keeling, Lubor Kostal, Alistair B. Lawrence, Michael T. Mendl, Mara Miele, Ruth C. Newberry, Peter Sandøe, Marek Špinka, Alex H. Taylor, Laura E. Webb, Laura Whalin, Margit Bak Jensen

**Affiliations:** ^1^ Institute of Animal Welfare Science, University of Veterinary Medicine Vienna, Vienna, Austria; ^2^ Biosciences Institute, Newcastle University, Newcastle upon Tyne, UK; ^3^ Herbivores, Université Clermont Auvergne, INRAE, VetAgro Sup, Saint-Genès-Champanelle, France; ^4^ Department of Veterinary and Animal Sciences, University of Copenhagen, Frederiksberg, Denmark; ^5^ University of Oslo, Oslo, Norway; ^6^ Animal Husbandry & Ethology, Albrecht Daniel Thaer-Institute of Agricultural and Horticultural Sciences, Faculty of Life Sciences, Humboldt-Universität zu Berlin, Berlin, Germany; ^7^ Department of Culture and Learning, Aalborg University, Aalborg, Denmark; ^8^ Department of Sustainable Agricultural Systems, BOKU University, Vienna, Austria; ^9^ Department of Applied Animal Science and Welfare, Swedish University of Agricultural Sciences, Uppsala, Sweden; ^10^ Centre of Biosciences, Slovak Academy of Sciences, Bratislava, Slovakia; ^11^ Scotland’s Rural College (SRUC), Edinburgh, UK; ^12^ The Roslin Institute, Easter Bush Campus, Midlothian, UK; ^13^ Animal Welfare and Behaviour Group, Bristol Veterinary School, University of Bristol, Langford, UK; ^14^ School of Geography and Planning, Cardiff University, Cardiff, UK; ^15^ Department of Animal and Aquacultural Sciences, Faculty of Biosciences, Norwegian University of Life Sciences, Ås, Norway; ^16^ Department of Veterinary and Animal Sciences and Department of Food and Resource Economics, University of Copenhagen, Copenhagen, Denmark; ^17^ Department of Ethology and Companion Animal Science, Faculty of Agrobiology, Food and Natural Resources, Czech University of Life Sciences, Prague, Czechia; ^18^ ICREA, Barcelona, Spain; ^19^ Institut de Neurociències, Universitat Autònoma de Barcelona, Barcelona, Spain; ^20^ School of Biological Sciences, University of Canterbury, Christchurch, New Zealand; ^21^ Animal Production Systems group, Department of Animal Sciences, Wageningen University and Research, Wageningen, The Netherlands; ^22^ The Norwegian Veterinary Institute, Ås, Norway; ^23^ Department of Animal and Veterinary Sciences, Aarhus University, Tjele, Denmark

**Keywords:** animal emotion, flourishing, good life, good welfare, happiness, pleasure

## Abstract

The concept of animal welfare is evolving due to progress in our scientific understanding of animal biology and changing societal expectations. Animal welfare science has been primarily concerned with minimizing suffering, but there is growing interest in also promoting positive experiences, grouped under the term positive animal welfare (PAW). However, there are discrepancies in the use of the term PAW. An interdisciplinary group arrived at a consensus that ‘PAW can be defined as the animal flourishing through the experience of predominantly positive mental states and the development of competence and resilience. PAW goes beyond ensuring good physical health and the prevention and alleviation of suffering. It encompasses animals experiencing positive mental states resulting from rewarding experiences, including having choices and opportunities to actively pursue goals and achieve desired outcomes’. The definition also considers individual and species-specific differences. It provides a framework for researchers to investigate PAW and thereby generate innovative, informative and reproducible science. Studies of PAW can contribute to a richer picture of an animal’s life and may elucidate the biological foundations of happiness. The definition creates opportunities to inspire scientific progress in animal biology and to align animal care practices, legislation and markets with societal expectations.

## 1. Background

Animal lives are characterized by both positive (e.g. pleasant or rewarding) and negative (e.g. unpleasant or aversive) experiences. Whilst minimizing negative experiences and associated harms may be the more urgent and prioritized response of animals, positive experiences and their associated benefits are likely to be crucial for their welfare.

Since the emergence of modern animal welfare science, the primary focus has been on the minimization of suffering linked to negative experiences such as pain, fear and distress. Indeed, scientific evidence that an animal species has the capacity to feel pain or fear has driven changes in animal welfare legislation (e.g. fish: [1]; cephalopods and decapods: [2]). Nonetheless, minimizing suffering is insufficient to promote animal welfare because an absence of suffering does not mean that all the proclivities of an animal are being fulfilled, and in particular it overlooks the potential benefits of positive experiences in life [3–6].

The view that positive experiences are important to an animal probably aligns with how most members of the public conceptualize animal welfare. Lay people generally associate animal welfare with the provision of opportunities for positive experiences, in addition to presupposing that animals should not experience pain or suffering [7,8]. Animal-based industries recognize these consumer expectations, and commercial labels advertise products from ‘happy animals’ or from animals given a ‘good life’ (e.g. laying hens: [9], sheep: [10], dogs: [11], dairy cattle: [12]). However, science-based evidence is needed to validate these claims [13].

The emerging concept of positive animal welfare (PAW) places an explicit focus on positive aspects of animal lives. The concept of PAW offers opportunities to improve animal welfare through a better understanding of situations and mechanisms leading to positive experiences and their short- and long-term benefits to animals. However, PAW lacks an explicit scientific definition, having been used to refer to phenomena ranging from positive emotions and engagement with the environment to a good life and overall happiness in animals (reviewed in [14,15]). As a result, the topic of PAW consists of a variety of concepts and ideas emerging from different fields including behavioural biology, evolutionary biology, cognition, neuroscience, psychology, ethics and philosophy.

Despite the intuitive appeal of this concept, the lack of a definition is one factor that may hamper scientific progress in this area. A clear definition of PAW would safeguard against the dilution of its meaning, which could jeopardize its usefulness, as has been seen in other areas of science. For example, 43 discrete definitions of empathy have been identified [16], and this inconsistency in definition has compromised the comparability of studies, led to mismatches between research and treatment and education programmes and resulted in variable therapeutic effectiveness. A consensus on the definition would help to develop consistent scientific approaches that advance our understanding of PAW.

## 2. A definition of positive animal welfare

A scientific network entitled ‘Laying the foundations for positive animal welfare’ (LIFT) has been established to advance the scientific understanding of PAW in a cohesive and rigorous manner [17] (https://LIFTanimalwelfare.eu/). This network comprises more than 330 scientists from 42 countries. A fundamental goal has been to create shared definitions of key concepts of PAW. As a first step towards this aim, we convened to seek consensus on the definition of PAW as an interdisciplinary working group within LIFT.

The process to establish the definition and reach consensus was as follows. The authors were invited by the first and last authors to participate in a survey about PAW, followed by a 2-day discussion, employing small-group brainstorming, whole-group deliberation and voting to achieve consensus. The group was composed of academic scholars with expertise in biological, animal, biomedical, social science and humanities research and represented individuals actively engaged in research relevant to PAW. The group was gender-balanced and diverse in age and positions (i.e. postdoctoral researchers to professors). All were employed at European academic institutions.

We achieved a consensus definition among ourselves with relevance to non-human animals in homes, farms, laboratories, zoos, and the ‘wild’, but acknowledge that members of other disciplines, cultures, ways of knowing (e.g. [18]), professional occupations or those aiming to use models to better represent non-human animals in democratic decisions (e.g. [19]) could propose different themes for PAW.

This article explains our resulting definition (box 1) which contains important keywords that are expanded upon below.

Box 1. **Positive animal welfare definition.**

‘Positive animal welfare is defined as the animal flourishing through the experience of predominantly positive mental states and the development of competence and resilience. Positive animal welfare goes beyond ensuring good physical health and the prevention and alleviation of suffering. Positive mental states result from rewarding experiences, including having choices and opportunities to actively pursue goals and achieve desired outcomes, according to species-specific and individual capabilities. Genetic, developmental and experiential factors (e.g. pre-natal, early life, environmental) contribute to individual differences in the ability to achieve positive animal welfare. Positive animal welfare can be assessed using animal-based indicators and can be evaluated over different timescales, thereby contributing to the lifetime picture’.

*‘Positive animal welfare is defined as the animal flourishing through the experience of predominantly positive mental states and the development of competence and resilience’*.

In humans, *flourishing* is a composite concept that typically includes positive emotions, life satisfaction or happiness, engagement, meaning and purpose or a sense of achievement, fulfilling and supportive social relationships and resilience [20,21]. Flourishing links to mental and physical wellbeing [22,23]. Other authors refer to the achievement of one’s full potential [24]. Animals may experience attributes linked to flourishing such as affective happiness, defined as a long-term state reflective of how an animal feels most of the time [25], flow as a state of complete absorption in an intrinsically rewarding activity [26], and supportive and fulfilling social relationships [27]. Thus, flourishing is an aspirational goal for scientists and animal caretakers to work towards understanding and enabling in animals. Research is needed to identify (likely species-specific) features, scientific methods and valid indicators of flourishing in animals.

*Positive mental states* are often referred to as positive affective states [28], which comprise both short-term ‘emotions’ that are elicited by a stimulus or event, and longer-term ‘moods’ that are dissociated from a specific event [29]. We chose to refer to them under the umbrella term of mental states, encompassing both affective (i.e. valenced) and cognitive processes. As in the fields of human psychology and psychiatry, the study of animals’ mental states has tended to focus on the negatives, such as fear, distress and pain, or mood disorders such as clinical depression [30,31]. Nevertheless, interest in searching for and evaluating positive mental states, such as joy and relaxation, has been rising (e.g [1,4,32–36]). Animals’ mental states may be indicated by changes in behaviour, cognition and physiology [37]. For instance, optimistic cognitive judgement biases, hypothesized to reflect positive mental states [38], have been reported across taxa ranging from mammals to eusocial insects [39,40]. In humans, positive emotions are related to self-declared happiness levels [41,42]. We included the adverb *predominantly* in this definition to specify that this state can be interspersed with negative mental states. The way in which positive and negative affective states interact to influence welfare states is an important question that warrants research [43].

*Developing competence* can be intrinsically rewarding [26,44] and improve an animal’s ability to deal with a range of challenges and enhance its capacity to utilize opportunities. Animals may acquire competence through, for example, exploring their surroundings [27], performing play behaviour [45] and practising decision-making [46]. In this process, animals may develop behavioural, cognitive and emotional competences [44], along with physiological and immune competences [47,48], that can be useful in the future. Competence relates to concepts found in the animal literature like agency, which can be broadly defined by the capacity for inner motivated behavioural engagement with the environment [44,49,50], the effectiveness concept that combines motivations leading to desirable outcomes, information gathering and being in control [51], or the dynamic animal welfare concept [52]. Similarly, the broaden-and-build theory in the human literature postulates that positive mental states may encourage individuals to play, explore and learn, thereby building competence [53].

*Resilience* is commonly defined as an animal’s capacity to maintain or regain healthy physical and mental functioning in the face of environmental disturbances [54,55]. This can occur by being minimally encumbered by challenging events (i.e. the magnitude of the response is lowered, also referred to as robustness [56]), or being better able to recover from them (i.e. the duration of the response is shortened). However, resilience should not be used to justify the infliction of pain and other forms of suffering on animals by arguing that a practice is acceptable because resilient animals are less affected by it.

These aspects (positive mental states, competence, resilience) interact in complex ways, and how they each contribute to PAW could be the subject of further research, as has been done in humans (e.g. [57]).

*‘Positive animal welfare goes beyond ensuring good physical health and the prevention and alleviation of suffering’*.

*Good physical health* is not sufficient for an animal to experience PAW. Though conditions may ensure good physical health for animals, they may deprive them of other aspects important to their welfare. For instance, it may be easier to limit pathogen exposure and physical injuries by keeping animals in barren cages or pens but such spatially and sensorily restrictive environments thwart animals from performing certain behaviours, which can lead to frustration [58–60]. Furthermore, restrictive environments often deprive animals of opportunities to perform behaviours that are suggested to have rewarding properties, such as affiliative social interactions [27] and play behaviour [61]. Where individual or population capabilities are impaired, for example through artificially selected characteristics, the ability to experience PAW may also be hindered [62,63].

We included *beyond […] the prevention and alleviation of suffering* in the definition because PAW cannot be achieved solely by the absence of negative states such as pain and distress. Though studies have suggested that the reduction or avoidance of a negative stimulus may elicit transient positive mental states (e.g. relief: [64]), contexts and goals related to the removal of suffering does not qualify as PAW, despite being important for welfare improvement. For example, reducing risk factors or providing treatment for lameness may decrease suffering (i.e. minimize the pain), but does not result in PAW.

*‘Positive mental states result from rewarding experiences, including having choices and opportunities to actively pursue goals and achieve desired outcomes, according to species-specific and individual capabilities’*.

*Rewarding experiences* refer to acquiring or being exposed to resources and situations that stimulate behaviours motivated by wanting or reinforced by liking [65]. These aspects of liking and wanting are important mechanisms for proximate behavioural control [66], relevant to the perspective that it is important to animals to ‘have what they want’ [67], and developed for instance in the concept of positive affective engagement [68]. Fraser & Duncan [3] postulated that it is the pleasure inherent in some behaviours that motivates the animal to perform these when the opportunity arises and the cost of doing so is low.

*Having choices* has been associated with positive changes in animal behaviour and physiology (e.g. [69,70]). Having *opportunities to actively pursue goals and achieve desired outcomes* emphasizes that animals should be offered circumstances enabling them to engage in activities that are intrinsically rewarding to them [26,44,68]. Nevertheless, this may need to be accompanied by a successful result in order to be rewarding [28]. *According to species-specific and individual capabilities* highlight the need to consider differences in species and individual sensory worlds, capacities and needs [71,72]. That is, a situation that can elicit PAW in one species or individual may be ineffective or even detrimental in another.

*‘Genetic, developmental and experiential factors (e.g. pre-natal, early life, environmental) contribute to individual differences in the ability to achieve positive animal welfare’.*

There may be different individual predispositions to experience PAW. *Genetic* differences can play a role. For example, dogs varying in personality and breed can differ in their sociability [73], and rats from anxious strains show reduced social play and pleasure-related ultrasonic vocalizations [74]. Individual differences may also result from *developmental* factors during ontogeny, such as developmental plasticity [75], prenatal stress [76,77], experiences during sensitive periods [78] or epigenetic changes [79]. *Experiential factors* such as previous exposure [80–82], or repeated encounters with negative events, i.e. frequent and/or unpredictable stressors, are risk factors for depression [83] and affect individuals’ sensitivity to later events. For example, rats work harder for a preferred sucrose solution after a negative event [84] and animals in a bored state are more responsive to stimulation [85]. Conversely, anhedonia may reduce animals’ motivation to engage in an activity or their ability to derive pleasure from it [86]. All these factors, singly or in combination with each other, contribute to *individual differences* that can modulate an animal’s willingness to engage in an activity or utilize resources provided to them to experience PAW (e.g. [87,88]).

*‘Positive animal welfare can be assessed using animal-based indicators and can be evaluated over different timescales, thereby contributing to the lifetime picture’.*

*Animal-based indicators* provide a closer reflection of the animal’s PAW state than resource- or management-based indicators [89], although some resource- or management-based indicators can still be predictive of welfare states if they are strongly related to animal-based indicators. A few indicators of PAW have already been proposed [90]. Indicators are often context- and species-specific and require caution on their validity and interpretation [14,15,91].

We mention *different timescales* to reflect that PAW assessment can be conceptualized over different periods, from short-term positive experiences to the longer-term, even lifelong, mosaic of experiences and attributes that allow an individual animal to flourish. The long-term aspect of PAW links it to concepts such as quality of life [92], animal happiness [25] and other assessments of the *lifetime picture* and whether the animal has experienced a good life [93].

## 3. Implications

There are several reasons why this definition of PAW is important. By outlining a consensus position, this definition provides a framework for focusing and aligning research questions in order to generate innovative, informative and reproducible science in this area. While not yet operational, establishing a conceptual (working) definition at this early stage will be beneficial in guiding research, as has been seen in other fields (e.g. pain: [94] and play behaviour [95]).

PAW offers a different lens through which to understand the lives of animals. Even when aiming for environmental enrichment, a goal seemingly oriented towards enhancing positive animal experiences, results have been primarily measured in terms of reductions in negative outcomes such as injuries, distress and other negative mental states. For instance, the incidence of tail-biting, an abnormal and injurious behaviour in pigs, was reduced by the provision of 20 g of straw per pig per day [96]. However, the time pigs spent rooting and manipulating straw continued to increase when increasing amounts of straw were provided up to approximately 250 g of straw per pig per day, suggesting that this amount was necessary to satisfy the pigs’ behavioural need to explore when housed indoors on a concrete floor [97]. Another example is the amount of space dairy calves need when kept in indoor pens. In a recent scientific opinion on calf welfare, EFSA recommends 3 m^2^ per calf to avoid resting problems (i.e. discomfort due to the inability to lie or rest comfortably), but 20 m^2^ per calf to allow the full expression of play behaviour, which is rewarding for the animal [98]. Thus, approaching questions from a positive standpoint can lead to different results, as it turns the question from how to avoid problems to how to satisfy the animals, thereby ‘raising the bar’ for animal care [91].

This definition is aspirational and progressive, as it incorporates concepts such as flourishing, positive mental states, resilience, competence, choice and active pursuit of goals. It creates an exciting opportunity for researchers to advance our understanding of wellbeing in both humans and non-human animals by developing and integrating methods from both natural and social sciences. In this way, this definition can inspire research leading to new methods for assessing PAW. By catalysing the development of new methods, this definition could stimulate empirical approaches to investigate evolutionary pathways and thereby improve our understanding of the biological foundations of human happiness. This has the potential to impact multiple areas of research, including comparative psychology, behavioural biology, evolutionary biology, animal ethics, consciousness science and philosophy.

The progressive nature of this definition is limited by current scientific methods and empirical evidence. First, we included flourishing despite the lack of clear scientific conceptualization and operational approach (even in humans: [21,99,100]). We consider that this should stimulate scientific research and discussion of this concept rather than discourage the use of this term. Second, the reference to ‘experiences’ implies sentience, i.e. the capacity to consciously experience positive and negative mental (affective) states including emotions, moods and valenced sensations such as pain and pleasure, and hence to have subjective ‘feelings’ [101,102]. Though these states are key determinants of an animal’s welfare [103], they are inherently subjective and most researchers agree that it is not currently possible to measure them directly [104]. Nevertheless, there is a prevailing scientific opinion that animals of various taxa have the capacity for sentience, i.e. that they can consciously experience negative or positive feelings such as pain or pleasure (e.g. [32,104–107]). Yet, this is contested by some scientists (e.g. [108,109]), pointing out that motivational and affect-like processes can occur without being consciously felt, including in humans [110]. As we still do not know which species are likely to be sentient, we propose that the ‘precautionary principle’ should be used [111]. If an animal species has the capacity to feel pain, it is difficult to conceive how this species could be devoid of the capacity to experience positive mental states like pleasure [3], and therefore species that currently fall under animal welfare considerations also deserve PAW considerations.

The concept of PAW has the potential to frame and stimulate novel research, thereby contributing to a richer and more complete picture of animal welfare. This definition is complementary to existing definitions of animal welfare (e.g. [112]), yet aligns more closely with the prevailing view of citizens that animals deserve a good life [7,8]. Therefore, at a societal level, this definition creates an opportunity, perhaps even pressure, for animal care practices, legislation and markets to mirror how society expects animals to be kept and treated. This definition provides a starting point and may need to be updated as research progresses based on emerging knowledge, international and interdisciplinary perspectives and input from stakeholders such as animal advocates, indigenous groups and industry representatives. Ultimately, we hope that this definition helps to focus research and inspire scientific progress for the benefit of both animals and humans.

## Data Availability

This article has no additional data.
